# Stress and self-esteem in young high school students

**DOI:** 10.1192/j.eurpsy.2023.1086

**Published:** 2023-07-19

**Authors:** K. Chiha, D. Ben Touhemi, M. Chaabane, J. Boudabous, W. Kammoun, I. HadjKacem, H. Ayadi, K. Khemakhem, Y. Moalla

**Affiliations:** Child Psychiatry, Hedi Chaker University Hospital, Sfax, Tunisia

## Abstract

**Introduction:**

The course of adolescence is marked by feelings of insecurity, vulnerability and can be accompanied by the emergence of several mental health problems.

Having a good self-esteem brings many benefits such as security, well-being and a strong sense of confidence. Low self-esteem is often accompanied by psychological distress such as stress.

**Objectives:**

To assess the level of stress and self-esteem in young high school students and to identify the risk factors associated with low self-esteem in these adolescents.

**Methods:**

This was a cross-sectional, descriptive and analytical study conducted among a sample of adolescents randomly collected in 6 schools in the region of Sfax-Tunisia, during the month of February 2022. The level of stress was assessed using the Lovibond Depression Anxiety Stress Scale (DASS-21) and self-esteem by the Rosenberg Self-Esteem Scale, both scales are validated in Arabic.

**Results:**

We collected 396 adolescents. The mean age was 16.65+/-0.897 years and the sex ratio was 0.82.

Of these adolescents, 102 had stress symptoms according to the DASS-21 scale, i.e. 26% of the sample. Stress was severe to extremely severe in 37.2% of cases.

Low to very low self-esteem was found in 65.7% of cases compared to 14.7% with high self-esteem.

In addition to the association with high levels of stress in these adolescents (p=0.002), low self-esteem was associated with other psycho-social factors such as intra-family relationship problems (p=0.014), a history of repeating a year (p=0.026), low to average school performance (p=0.027) and behavioural problems in the school environment (p=0.032).

**Conclusions:**

These results suggest that the association of stress with certain psycho-social factors helps the deterioration of self-esteem in adolescents and vice versa.

Having high self-esteem may protect the individual from psychological vulnerabilities such as stress and help him/her to cope with them.

**Disclosure of Interest:**

None Declared

